# Back to Babies: Reducing Documentation Time in the NICU

**DOI:** 10.1055/s-0044-1782531

**Published:** 2024-03-20

**Authors:** Shama Y. Patel, Rebecca S. Rose, Emily C. Webber

**Affiliations:** 1 Division of Neonatology, Nationwide Children's Hospital, Columbus, Ohio, United States; 2 Division of Clinical Informatics, Nationwide Children's Hospital, Columbus, Ohio, United States; 3 Department of Pediatrics, The Ohio State University College of Medicine, Columbus, Ohio, United States; 4 Division of Neonatal-Perinatal Medicine, Department of Pediatrics Riley Hospital for Children, Indiana University School of Medicine, Indianapolis, Indiana, United States; 5 Department of Pediatrics Riley Hospital for Children, Indiana University School of Medicine, Indianapolis, Indiana, United States

**Keywords:** electronic health records, documentation, neonatal intensive care unit, workflow

## Abstract

**Background** There is no accepted best practice for generation and content of daily progress notes in the neonatal intensive care unit (NICU).

**Objectives** This study aimed to implement a consistent documentation standard process for a neonatology provider group at a level IV tertiary care NICU. The primary aim was to improve timeliness of daily progress note completion. Secondary aims were to maintain or improve clinician satisfaction, reduce variability, and reduce attending neonatologist electronic medical record (EMR) documentation tasks.

**Methods** We formed a work group including advanced practice providers (APPs) and physicians from the NICU that met over 6 months to define the ideal NICU documentation content, map the workflow for documentation, identify gaps in EMR content, and create solutions for each gap. Baseline assessment included a change readiness survey to identify barriers to workflow change and a review of neonatologist signature timestamp to determine time to note completion. Twenty random progress notes were sampled weekly for 6 months prior to implementation of new workflow as well as 6 months postimplementation. Average time to note completion was compared in the pre- and postintervention groups.

**Results** In total, 962 notes were sampled, 481 each in the pre- and postintervention states. Twenty neonatologists were captured in the preintervention state, 24 in the postintervention state, 18 were captured in both samples. Final note completion time mean improved from 10 hours and 32 minutes (from starting note to final sign) to 8 hours and 40 minutes ( *p* < 0.01). Those sampled in both epochs improved from 10 hours and 6 minutes to 8 hours and 30 minutes ( *p* < 0.05).

**Conclusion** Progress notes generated by neonatologists are completed earlier than those generated by an APP with a Neonatologist addendum. Specialty-specific education and training are critical to high satisfaction in large EMR workflow transitions.

## Introduction

Over the last decade, the Health Information Technology for Economic and Clinical Health Act of 2009 rapidly accelerated Electronic Medical Record (EMR) adoption, 1 altering physician documentation from handwritten notes to typed documentation in the EMR.

Though physicians receive tremendous amounts of education and training on many facets of the work they do, interaction with the EMR and documentation are areas with little specific guidance provided and remain without best practice standards. Recently, some have proposed standards to address this gap 2 but historically the general structure of the Subjective, Objective, Assessment, Plan (SOAP) note as first described by Weed 3 in 1968 is the only universally accepted structure for progress note generation and this provides a basic framework that is open to interpretation by each individual physician, their specialty, or their local documentation culture.

This lack of standard workflow is magnified in the neonatal intensive care unit (NICU) with patients who are highly complex with long lengths of stay resulting in progress notes that are bloated and become a data repository for the entirety of the patient's stay. The simple act of visualizing, accessing, and reviewing such a massive amount of data are difficult. 4 Developing a progress note standard in the NICU poses unique challenges that are both institutional and technological, including consideration of the maternal–infant dyad, logistical challenges in dosing related to patient size, and their prolonged lengths of stay. 5 Few data exist regarding the efficacy and usefulness of NICU progress notes or their contents beyond work in computerization of NICU progress notes that resulted in time-saving for clinicians, increased legibility of notes, improved standardization of note layout and terminology. 6 Subsequent work found that a multidisciplinary approach to create and implement an accepted progress note documentation standard in the NICU can improve documentation, avoid provider dissatisfaction, and increase hospital payments. 7 Interventions that include both standardized templates and education have been found to improve note quality, decrease their length, and allow inpatient progress notes to be completed earlier in inpatient medicine services. 8

In our NICU, progress notes were generated in a multiauthor process. The initial template noted was created by an advanced practice provider (APP), once completed this was sent to the neonatologist for a free-text addendum and final signature. This multiauthor, interdependent workflow resulted in delays to note completion because the neonatologist could not addend and sign the note until the APP-generated note was finalized. Progress notes cannot fulfill their primary purpose as a tool for communication with other providers and a means for documenting important events and plans for the day when not completed in a timely manner. 9

## Objectives

Our primary aim was to improve timeliness of daily progress note completion and successfully adopt a system-wide EMR documentation process within the NICU. Our secondary aim was to maintain clinician satisfaction with the new documentation standard.

## Methods

The Riley Hospital for Children NICU is a level IV unit that has the capacity to hold 60 newborns. A Cerner© EMR is utilized which serves the comprehensive health system across the state (ambulatory clinics, surgery centers, adult hospitals, etc).

The project was divided into four sections: workflow and content assessment, workflow design and change management, training, and implementation.

The first step was establishment of a workgroup that included APPs and physicians from the NICU, this group functioned as a focus group and provided insights into the accepted practices, identified important themes in workflow analysis, potential obstacles and solutions to implementation of a new EMR workflow.

### Progress Note Content Assessment

**Fig. 1:**
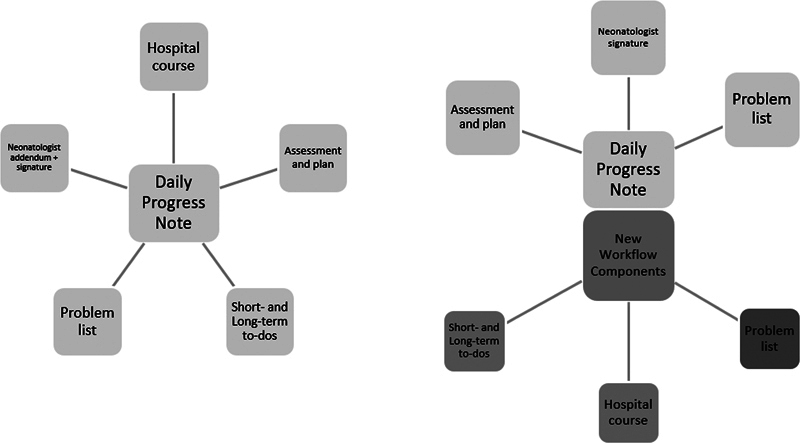
The diagram on the left illustrates the components that were found to be within the progress note in the preintervention workflow. The diagram on the right illustrates the new workflow that provided spaces outside the progress note for critical patient information.

Our review found that the NICU daily progress note contained the following elements ( Fig. 1 ):

- An assessment and plan for the day
- A hospital course, a longitudinal patient history up to the current point in time
- A problem list that was entered in individual fields
- Short- and long-term tasks interspersed throughout different sections of the note
- An attestation or addendum with an attending signature

### Workflow Assessment

#### Clinical Coverage

Most patients in the NICU are cared for by APPs supervised by a neonatologist. Resident physicians infrequently provide clinical coverage in this unit and were excluded from evaluation. Day team physicians provide coverage from 8:00 a.m. until 4:00 p.m. and are responsible for daily progress note generation.

#### Progress Note Generation

**Fig. 2:**
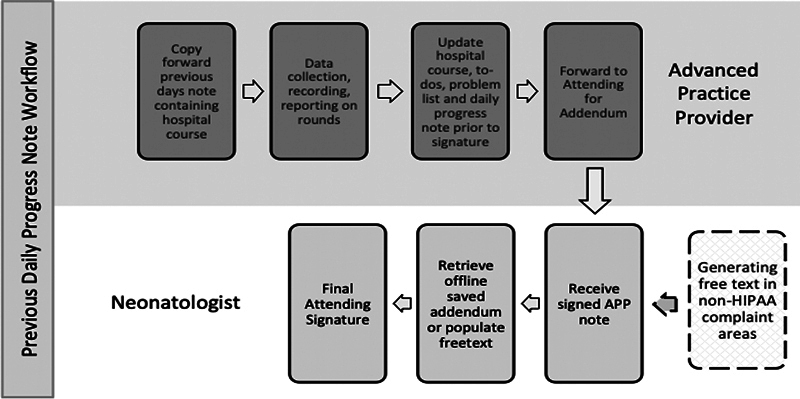
This workflow diagram illustrates the previous documentation process. The note generation process starts at the top left and progresses through the APP workflow until that is completed and is forwarded to the attending Neonatologist for final signature and completion. APP, advanced practice provider.

Within the stakeholder group, we established that the progress note was initiated by the APP by using the copy forward function that duplicated the entirety of the previous day's note ( Fig. 2 ). The APPs would then review laboratories, imaging, and other pertinent data from the EMR and subsequently present on rounds. After rounds, the APP would update the hospital course which was housed within the progress note and then update the typical sections of the progress note including the problem list, assessment, and plan. This APP-completed document was sent to the attending neonatologist for a final addendum or attestation to the note, that was either typed directly into the EMR, or by copy/paste from an offline word processing document which was neither secure nor Health Insurance Portability and Accountability Act (HIPAA)-compliant. This completed the progress note for the day.

#### Areas of Improvement

The workgroup identified lack of timely cohesive daily clinical narrative, redundant work, need for continuous contribution to the hospital course, and security risk (including HIPAA) that occurred by documentation content generation offline as the areas for improvement within the documentation workflow.

### Workflow Redesign and Change Management

The NICU workflow review and redesign preceded a system-wide EMR adoption of a new documentation workflow. A large organizational change was underway that transitioned documentation workflows from a traditional template-based electronic documentation process to one where components of required documentation were universally present on the screen and available for editing and transformation to a note when ready.

This EMR user interface change was implemented at an organizational level and was mandatory, given this the NICU workgroup aimed to leverage organizational project support for the transition to develop a new documentation process and specific tools that fit the needs of our unique population.

The workgroup also developed guidelines to facilitate efficient note writing. We defined the purpose of the daily progress to serve as a record for the clinical decision-making that occurred on rounds. Limiting the content to this snapshot in time allowed the neonatologist to write, sign, and complete the note any time after rounding on that patient and emphasized prioritization of simplified and clinically relevant patient information. We also concluded that the progress note should follow the accepted SOAP convention with a concise plan by systems.

We found that in the previous state progress notes held many important pieces of patient history, but these were not necessary components of a progress note. In the new workflow these components—to-dos and the hospital course—were moved to a separate continuously accessible and editable portion of the EMR but out of the signed daily progress note ( Fig. 1 ). These components were allocated specific places in the EMR in the new workflow to ensure all information about a patient was still accessible and safely stored. By parsing out the nonprogress note tasks out of the signed documentation workflow and shifting this historical recordkeeping to nonsignature-based fields the APP workflow and Neonatologist workflow were separated allowing for a single-author progress note workflow.

**Fig. 3:**
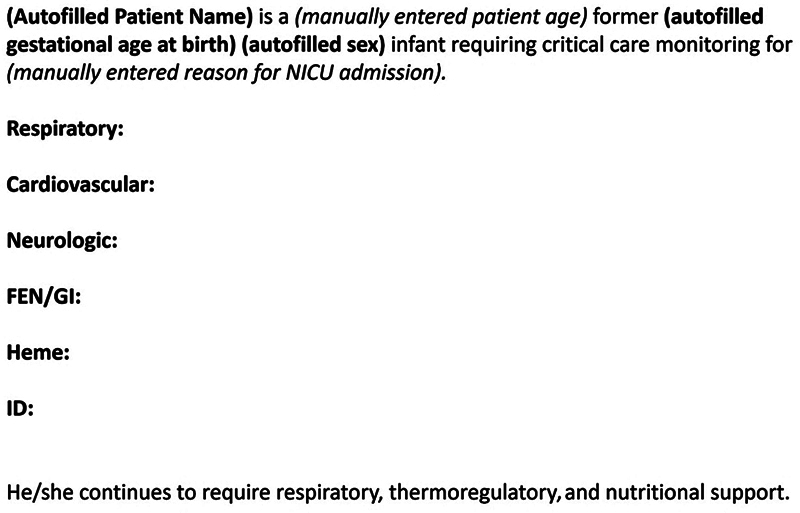
Example of text that could be generated via system shortcut that included subheadings by body system with fluids, electrolytes and admission and gastrointestinal combined abbreviated to “FEN/GI.”

To promote standardization, globally available autotexts were created with the help of the organizational project team that included the preferred formats for all documentation areas ( Fig. 3 ). These “dotphrases” or “macros” streamlined other content generation areas by providing a normal newborn exam and autopopulating certain patient information directly from the chart. The functionality of the new embedded, interactive note generation workflow would allow for selected “tagging” of elements through chart review that could be pulled into the final documentation to further reduce transcription error and duplication of information.

### Training

#### Baseline Assessment

A change readiness survey of APPs, neonatologists, and fellows that was reviewed by the workgroup provided an understanding of barriers to modifying documentation workflow. One-third of respondents identified lack of training as their biggest concern, to address this a robust education plan was developed and executed prior to implementation.

#### Specialty-specific Education

A neonatology-specific EMR workflow training plan was developed and deployed by a physician champion 6 weeks prior to implementation of the new workflow, which included

- A step-by-step PDF guide that illustrated how to set up the new tools, how to use them for note generation, and where new designated areas for specific patient information would be
- A video supplement to the PDF guide with screen recording and voiceover for multimodal learning
- In person training for any individual or small group to walk through the PDF guide with hands-on instruction
- A test patient training environment to allow for fully immersive training and practice
- Just in time training for those requiring training or retraining prior to their clinical service time

This multipronged approach ensured that all providers received training prior to utilizing the new workflow, the in person trainings were attended by 24 providers.

### Evaluation of Pre- and Postimplementation

For 6 months prior to transition to the new workflow we reviewed neonatologist signature timestamps. Based on available resources, 20 random progress notes were sampled every Monday for convenience and the time of neonatologist signature was collected as well as word count of attending addenda. The same sampling mechanism was utilized for 6 months postimplementation of workflow and the neonatologist signature time was collected as well as word count of the subjective section of the note and the assessment and plan, as both these sections required generation of text. Time to completion was calculated by using 8:00 a.m. as the designated start time of the day. The time to completion in hours and minutes was converted to a decimal value (e.g., 10:30 to 10.5). Average time to note completion and word count was compared in the pre- and postintervention groups. A random effect was that some neonatologists were only sampled in one epoch, while others were sampled in both. Thus, we utilized an unpaired *t* -test to compare all data as well as a paired *t* -test for the paired data. Regression analysis was done to evaluate for the effect of years in practice as a neonatologist as well as gender on time to completion. Finally, a survey of satisfaction was sent out on a rolling basis to those who had been exposed to the new workflow.

## Results

### Preimplementation Survey

The initial change readiness survey had 59 responses (out of 72), an 82% response rate. Neonatologists were the biggest group of respondents ( *n* = 27, 46%), followed by APPs ( *n* = 25, 42%), and lastly neonatology fellows ( *n* = 7, 12%). The response rate by role was 93% of neonatologists, 69% of APPs, and 100% of neonatology fellows. The biggest concerns identified were lack of training (33%), too much time doing notes (26%), coding/billing mismatch (15%), none (12%), and other (14%). Twenty-seven percent of respondents were excited for the new workflow (16), 62% were nervous but hopeful (37), 3% were upset (2), and 7% marked other (4). The previous workflow included a plan by systems, and 77% of respondents voted to continue a systems-based plan.

### Postimplementation

In total, 481 notes were sampled in both the pre- and postimplementation phases for a total of 962 notes reviewed.

**Fig. 4:**
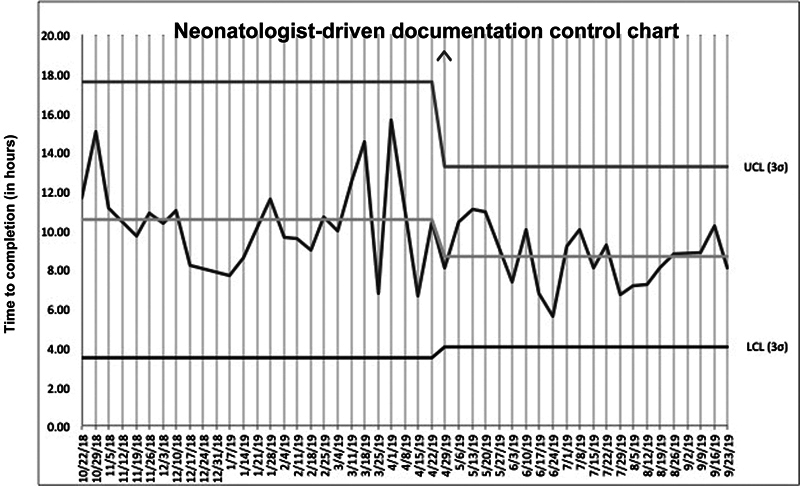
This control chart shows the shift in mean time to completion in the postintervention state. LCL, lower control limit; UCL, upper control limit.

**Fig. 5:**
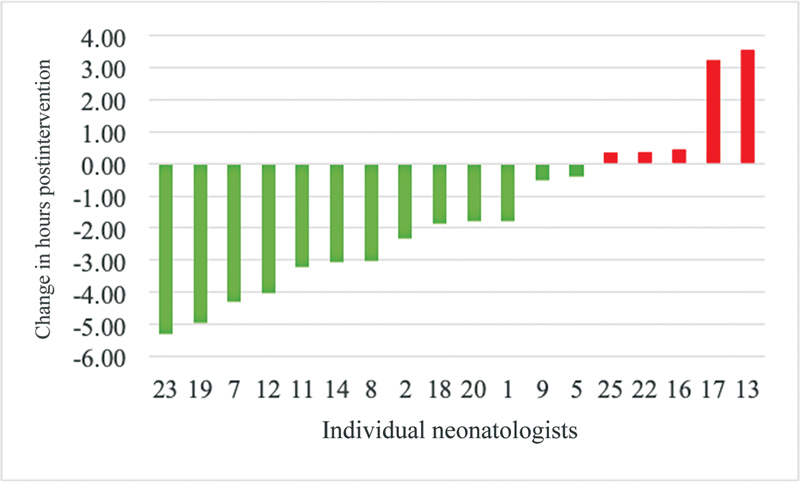
This bar graph shows the difference in time in hours of the neonatologists that were studied in both samples. Those in green were faster in the postintervention state while those in red were slower in the postintervention stage.

In the preimplementation period the mean time to completion was 10.32 hours with a standard deviation of 2.59 hours, in the postimplementation period the mean time to completion was 8.30 hours with a standard deviation of 2.22 hours. This was found to be significant mean difference ( *p* = 0.009; Fig. 4 ). For neonatologists in both samples the preimplementation mean time to completion was 10.10 hours with a standard deviation of 2.22 hours, in the postimplementation period the mean time to completion was 8.43 hours with a standard deviation of 2.19 hours, this was found to be significant ( *p* = 0.01). Of the 18 neonatologists captured in both samples 13 had earlier times to completion while 5 had later times to completion ( Fig. 5 ). Of those five, three had increases of less than 30 minutes to their overall documentation time.

In the preimplementation period the mean word count of the attending addenda was 149 with a standard deviation of 61 words, in the postimplementation period the mean word count of the subjective, assessment, and plan in was 191 with a standard deviation of 47 words. This was found to be a significant mean difference ( *p* = 0.013). For those captured in both samples the preimplementation mean for word count was 152 with a standard deviation of 63, in the postimplementation period the mean word count was 191 with a standard deviation of 53. This was found to be a significant mean difference ( *p* = 0.023). Regression analysis showed no significant impact of gender or years in practice in either time to completion or word count.

**Fig. 6:**
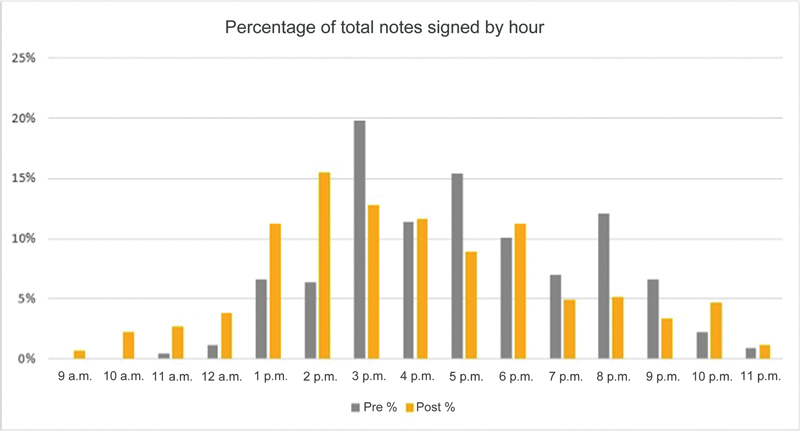
This graph shows the percentage of notes signed by hour in the pre- versus postimplementation state.

In the previous state the earliest time a note was signed was 11:52 a.m. and the latest signature time was 11:46 p.m. ( Fig. 6 ). Zero percent of notes were signed before noon, 34% between noon and 4 p.m., and 66% after 4 p.m. In the postimplementation period the earliest time a note was signed was 9:14 a.m. and the latest signature time was 12:15 a.m., the following day. Six percent of notes were signed before noon, 43% between noon and 4 p.m., and 51% after 4 p.m.

A satisfaction survey had 30 respondents with a 48% response rate. Twelve respondents were neonatologists and 18 were APPs. In total, 23.3% were very satisfied (7), 43.3% were satisfied (13), 6.7% were dissatisfied (2), and 0% were very dissatisfied.

## Discussion

We found that by utilizing NICU-specific workflows and leveraging direct authorship by the attending neonatologist, progress notes were completed earlier in the day. The primary reason for this was reduced workflow redundancy by condensing the progress note generation workflow from multiauthor to single author. This underscores the need to designate specific roles to unique users of the EMR to optimize efficiency and reduce duplicative work. Our study found significant time-saving from clearly defined workflow roles with marginal increase in individual documentation burden, illustrated by the significant time-saving for most neonatologists with a minimal increase in word count. Despite a statistically significant increase in content generation by neonatologists, the overall time to completion was reduced. We expected to see an increase in word count postimplementation because the preferred format included autotexts with subheadings by system included, creating a baseline word count already present prior to any content generation by the end user. Neonatologists who had word counts less than 50 in the previous state were utilizing a saved autotext to populate a standard attestation, meaning they were generating little to no free text at all in the previous state. Ultimately, this did not impact our primary outcome.

**Fig. 7:**
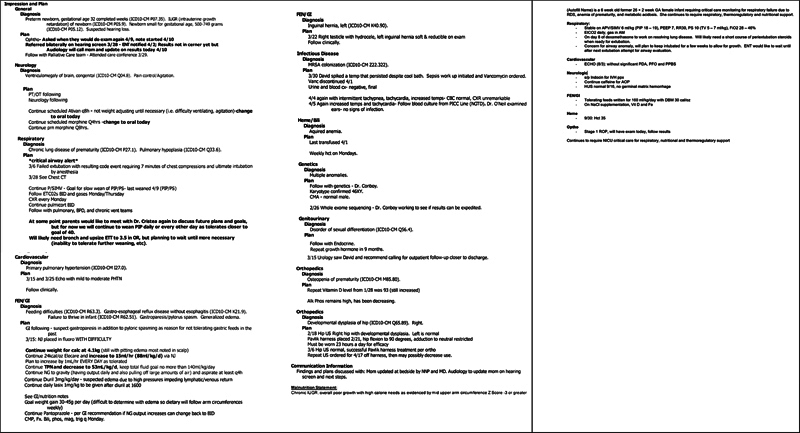
A side-by-side comparison of the structure of the assessment and plan portion of the note in the preintervention state (left) compared to a simplified structure in the postintervention state (right).

In the postimplementation state progress notes are improved in several ways. First, the NICU daily progress notes are more consistently updated and completed earlier in the day. Second, the progress note reemerged as a place to capture the clinical narrative for the day and resume its primary function as a communication tool amongst providers and a place to record important events of the day and the plan. 9 Timely and accurate progress notes can provide important and relevant details for other care team members including consulting physician teams, nursing, and other allied health professions aiming to understand the patient's status. By defining the major categories of information about these patients that existed and allocating specific places in the chart to hold that information we successfully separated an intertwined workflow while ensuring all patient information is still maintained. The APP–Neonatologist team could focus jointly on contributions to the hospital course, updating the problem list and even more time committed to patient care. In the previous state the APP-generated progress note relied heavily on a point and click template, but significant variability remained in the final product. Similarly, the attending free text addenda had no defined standard yielding significant variability in length and content but the use of a standard autotext-based template for both the progress note as well as the ongoing updated areas decreased variability in the new state ( Fig. 7 ). By eliminating the need for an addenda the risk that was posed by this information being generated in a non-HIPAA-complaint manner such as saving on the desktop of a shared computer or in a word processing program was completely eliminated. Finally, in the new workflow there was no longer a risk of information presented in duplicate or triplicate due to multiple points of data transfer and multiauthorship.

In addition to those improvements to the progress notes, our study supports a key finding that a robust specialty-specific education plan allows for maintenance in satisfaction even with large EMR transitions. This is bolstered by our survey results that found that the largest area of concern during this transition was lack of training in the new workflow. Physicians teaching other physicians during clinical work has been shown to improve physician efficiency 10 and we built our training program to mirror this type of environment. We illustrated that teaching done by a peer, with appropriate use of immersive training utilizing a test patient to simulate clinical work, maintained satisfaction with a new standard. This was key to successful implementation; training hours and the mastery that comes from them are a key component of EHR satisfaction. 11 In addition, we found this teaching model can be used to improve timeliness of note completion. As illustrated by Robinson and Kersey, 12 we found that timely, specialty-specific education was imperative to our successful EMR adoption. Although our survey was designed to meet the needs of this project, our respondents reporting satisfied or highly satisfied is very encouraging for this type of adoption process.

A specific strength of this project was a complete departmental transition to the modified workflow 3 months prior to the institutional decommissioning of the previous workflow. Although the previous workflow components were still available at the time of the department transition there were no progress notes generated in the previous format demonstrating full buy in from all participants. As noted previously, a key element to the success of this project was clinician-created individualized education. Though some general education on the new workflow was provided at the organizational level for all end users it was not specialty-specific. The Neonatology-specific teaching guide created by a physician champion was very important to the success of implementation and continued adherence to the standard. Many clinical documentation workflows rely on multiauthor notes via residents or other APPs and the solutions explored here could be generalized to other settings.

There are also some limitations of this work. One is the small sample size of the physician group studied; despite this we found a large effect in time to completion of notes. Also, EMR vendors are limited in the granularity of the data that can be reported 13 about time spent in documentation or time spent in discrete areas of chart review and that resulted in an inability to quantify time spent directly in documentation or in other areas of documentation that did not result in signed notes. Due to some of these limitations we used word count as a surrogate for effort spent in documentation, in the future, better metrics for this should be created and followed. In our study word count was further confounded by using “macros” and “dotphrases” that contributed a baseline word count prior to any end-user text generation. A final factor to consider in this specific implementation is that a large mandated organizational transition to the documentation workflows was being instituted and provided a natural opportunity for significant reassessment and reorganization of a well-established clinical and documentation workflow. While this provided some increased opportunity for change it did not allow for robust investigation and evaluation of all metrics that could be evaluated in the pre- and postintervention state and leaves room for further exploration in the future.

The usefulness of this work is multifaceted. Though other studies have illustrated the benefits of utilizing a standardized note template there has been little to no evaluation of what to include in this standardized note. 14 15 We found that a successful EMR transition can be achieved by a carefully constructed and delivered education plan. Despite concerns for increasing the documentation burden on individual neonatologists, the simplified workflow improved efficiency allowing most neonatologists to generate more content in less time. It has been a challenge to provide an objective measure of the decentralized work that proceeded this effort (writing addendums in Word and then cutting and pasting), which has made our other assessments important in demonstrating value and improved efficiency.

## Conclusion

The need for commonly accepted standards in progress note generation and format is paramount and each subspecialty may need to come to their own consensus to fit their individual needs 2 and our work is one example of this process. Further study is required to understand the quality of the notes postintervention for content and to evaluate if time efficiency in the new standard is maintained or if there was some impact of being evaluated at the time of initial implementation. Also, the significant time-saving yielded by transitioning authorship singularly to the neonatologist might suggest increased documentation efficiency by role and warrants some further study.

Large EMR transitions are inevitable in the era of fully EMR systems. Standardization of documentation practices is vital to maintain efficiency on an individual level and facilitate communication between the clinical care team. A robust education plan that is specialty-specific can mitigate provider dissatisfaction in transitions and increase timeliness in note completion.

### Future Directions

Continuing to identify and follow metrics to quantify provider work in the EMR presents an ongoing challenge but provides a significant area for future exploration. While documentation signature times can sometimes be more easily quantified, understanding time spent in reviewing and updating information that is not signed can also provide insight into the effort expended in the EMR and a way to identify areas for efficiency. Another interesting area of study will be evaluation of the benefits that emerge from reduced variation in documentation and EMR workflow practices. It is well established that adherence to guidelines and a reduced variation in clinical workflows improves care delivery and outcomes, similar benefits may emerge in this context as well. Finally, as we move into a more automated and data-driven environment, will this standardization further augment our ability to collect and analyze data and gain a deeper understanding of our patients.

## Clinical Relevance Statement

This study illustrates that large EMR transitions can be successful if appropriate training and education mechanisms are set in place and end user efficiency is improved.
